# Amino acid stable carbon isotopes in nail keratin illuminate breastfeeding and weaning practices of mother – infant dyads

**DOI:** 10.1007/s00726-024-03425-2

**Published:** 2025-01-30

**Authors:** Hana Salahuddin, Andrea L. Waters-Rist, Fred J. Longstaffe

**Affiliations:** 1 Anthropology, Western University, London, Canada; 2 Earth Sciences, Western University, London, Canada

**Keywords:** Amino acids, Isotope analysis, Breastfeeding, Weaning

## Abstract

Compound-specific stable carbon isotope analysis of amino acids (CSIA-AA) is widely used in ecological studies to analyze food-webs and is gaining use in archaeology for investigating past diets. However, its use in reconstructing breastfeeding and weaning practices is not fully understood. This study evaluates the efficacy of stable carbon isotope analysis of amino acids in early life diet reconstruction by analyzing keratin from fingernail samples of three mother-infant pairs during late gestation and early postpartum periods. Our results show that stable carbon isotope ratios (*δ*^13^C) of glycine, and to a lesser extent glutamate, effectively trace the onset of exclusive breastfeeding and the end of weaning in infants. We propose that glycine’s ‘conditionally essential’ metabolic pathway during infancy allows it to reflect maternal glycine *δ*^13^C, indicating breastmilk consumption. Subtle changes in glutamate *δ*^13^C likely result from its ‘non-essential’ status. Additionally, *δ*^13^C values of glycine and glutamate indicate maternal physiological and pathological stress due to catabolic effects such as gluconeogenesis. These findings have significant implications for ecological and archaeological research using CSIA-AA for dietary reconstructions. They highlight the need to understand how metabolic pathways affecting *δ*^13^C of amino acids may change over an individual’s lifespan or be altered due to various forms of stress.

**Supplementary Information:**

The online version contains supplementary material available at 10.1007/s00726-024-03425-2.

## Introduction

Stable isotope analysis (SIA) is a valuable tool for investigating past dietary practices, particularly between mothers and infants (Waters-Rist 2023). However, the interpretation of stable isotope data from bulk tissues (e.g., collagen from bones or teeth; keratin from hair or nails) can be complicated and uncertain due to the effect of factors like growth, disease, and physiological or nutritional stress (Fuller et al. 2004; Hobson et al. 1993; Waters-Rist and Katzenberg 2010). Unlike bulk tissue SIA, compound-specific isotope analysis (CSIA) examines individual amino acids (AA). Amino acids are the fundamental building blocks of proteins and their stable isotopic composition is influenced by the chemical nature of each individual AA and its unique metabolic pathway (Ambrose and Norr 1993; Choy et al. 2010). Some AAs are more indicative of the isotopic composition of diet, while others can provide insights into metabolic processes (McMahon and Newsome 2019), enabling researchers to distinguish between dietary and non-dietary isotopic changes. This pilot study is part of a larger project aimed at examining early childhood diet using CSIA-AA for modern and archaeological samples. This paper presents our analysis of compound-specific stable carbon isotopes (*δ*^13^C) for reconstructing early life diet via a longitudinal analysis of keratin from fingernail samples of three mother-infant dyads. We focus on specific AAs such as glycine, glutamate, and phenylalanine due to their distinct biosynthetic pathways, which provide unique insights into diet and physiological or pathological stress. Additionally, we investigate the application of a widely used spacing ratio between the *δ*^13^C of glycine (Gly) and phenylalanine (Phe) (Δ^13^C_Gly-Phe_) to discern terrestrial and aquatic protein diets.

Although the participant group is small, this study is among a few that focus on using CSIA-AA to exclusively examine maternal reproductive and infant feeding practices (e.g., Cheung et al. 2022; Harris 2020; Romek et al. 2013). To improve our understanding of CSIA-AA for reconstructing early childhood diets, this study delves into AA metabolic pathways and their influence on *δ*^13^C patterns during the late prenatal and early postnatal periods for mothers and fetuses/infants (i.e. 14 weeks *in utero* to 1.5 years of age). This investigation is vital because the body’s AA sourcing may shift from dietary intake during childhood to endogenous synthesis later in life, impacting stable isotope patterns (Jim et al. 2006; Wu et al. 2013). These findings are significant for CSIA-AA ecological research, as researchers seek to understand diets and trophic positions across ecosystems, despite limited knowledge of changes in AA routing over an individual’s lifespan.

## AA routing and metabolism

Amino acids are categorized as essential or non-essential based on their metabolic pathways. Essential amino acids (EAAs) like lysine, leucine, phenylalanine, and valine cannot be synthesized by the body and must be obtained from dietary protein sources. Since they are not significantly modified before incorporation into tissues, *δ*^13^C of EAAs (*δ*^13^C_EAA_) closely reflect dietary *δ*^13^C_EAA_. Non-essential amino acids (NEAAs), such as glutamic acid and alanine, can be synthesized from the body’s carbon pool, leading to isotopic fractionation (Ambrose and Norr 1993; Newsome et al. 2014). Since NEAAs require significant energy for biosynthesis, they may also be derived from dietary sources (Hesse et al. 2022). The balance between dietary routing and de novo synthesis affects carbon fractionation from diet to tissue. Some NEAAs can also act like EAAs under certain conditions. For instance, during infancy, when the body’s growth requirements are exceptionally high, some mammals may not produce sufficient amounts of glycine and proline, making these AAs conditionally essential (CEAA) (Jim et al. 2006; Wang et al. 2014; Wu et al. 2013). This occurs because the de novo synthesis of these AAs requires more energetically expensive steps compared to other NEAAs (Voet and Voet 1995). This is crucial during breastfeeding and weaning periods, highlighting the need to understand how metabolic changes in CEAAs influence *δ*^13^C and the interpretations of CSIA data.

### Glycine and other NEAAs in utero

Glycine is an important AA in mammals and other animals as it plays a role in metabolic regulation, anti-oxidative reaction, and neurological function (Wang et al. 2013). It can be endogenously synthesized from other AAs, namely (1) serine (Arnstein & Keglević 1956) and to lesser extents, (2) choline (Soloway and Stetten 1953) and (3) threonine (Dale 1978; Hartshorne and Greenberg 1964). Cetin’s (2003) study on the transport of AAs from the placenta to the fetus in normal and growth-restricted pregnancies found that the transfer rate of glycine from mother to fetus was significantly lower than other AAs. Based on this finding, the author suggested that glycine can be newly synthesized in the feto-placental unit itself without significant input from the mother. Similarly, studies have shown that glutamic acid is not significantly taken up from the maternal placental side but is very efficiently synthesized within the placenta and transported to the fetus (Malek et al. 1993; Schneider et al. 1979). Hence, it is assumed that the most important pathway for fetal NEAAs is de novo placental production with minimal uptake from the mother, while EAAs are directly routed from the maternal to fetal units of the placenta with little or no fractionation. To shed light on the poorly understood topic of fetal routing of glycine in humans, we investigate *in-utero* glycine *δ*^13^C (*δ*^13^C_Gly_) of mother-infant dyads.

### Glycine in diseased states and neonates

Several studies have shown that the amount of glycine synthesized in vivo is insufficient to meet the metabolic demands of an organism in a diseased state (Jackson 1991; Meléndez-Hevia et al. 2009; Wu 2010). Mild insufficiency of glycine is not life threatening; however, a chronic shortage may result in suboptimal growth, an impaired immune response, and other adverse metabolic and health effects (de Koning et al. 2003; Lewis et al. 2005). In birds, dietary glycine is essential for fetal (Jackson et al. 2002) and neonatal (Wu and Knabe 1994) development, since in early life they cannot synthesize sufficient glycine to meet requirements (Cetin et al. 1995; Paolini et al. 2001). Research on glycine routing in humans during the early stages of life remains limited, creating gaps in our knowledge concerning the use of *δ*^13^C_Gly_. Our investigation explores glycine metabolism during early life, seeking to determine whether it functions as a NEAA or a CEAA and how that may influence *δ*^13^C_Gly_. This knowledge would aid distinguishing and identifying possible physiological effects on *δ*^13^C_Gly_ and tracking breastmilk consumption more clearly.

### Applications of *δ*^13^C_Gly_

Previous studies using CSIA-AA for dietary reconstruction have primarily utilized *δ*^13^C_Gly_ to differentiate terrestrial and aquatic diets (Corr et al. 2005; Honch et al. 2012; Webb et al. 2018). Aquatic diets, both marine and freshwater, show higher *δ*^13^C_Gly_, correlating with higher trophic levels due to greater isotopic fractionation in marine ecosystems (Larsen et al. 2013). As phenylalanine *δ*^13^C (*δ*^13^C_Phe_) remains relatively unchanged between diet and consumer, Δ^13^C_Gly−Phe_ is used to distinguish aquatic from terrestrial protein diets. Corr et al. (2005) found that individuals consuming marine protein exhibited elevated Δ^13^C_Gly−Phe_ (12.0 ± 1.9‰) compared to those consuming C_3_ (5.1 ± 1.8‰) and C_4_ terrestrial protein (4.0 ± 1.6‰). However, the study focused on adult human and faunal bone collagen, leaving the effectiveness of this metric for nonadults or diseased individuals unclear due to the conditionally essential nature of glycine. Here, we apply Δ^13^C_Gly−Phe_ to mother-infant dyads to investigate its effectiveness in distinguishing diets during breastfeeding and weaning, and under various pathological or physiological conditions.

## Materials and methods

### Sample collection

Fingernails were chosen for analysis because they grow incrementally and have the potential to provide longitudinal dietary information if collected over time (e.g., Buchardt et al. 2007; Fraser et al. 2006; O’Connell et al. 2001; Williams and Katzenberg 2012). A total of 43 fingernail samples were collected from three mother-infant dyads before and after birth, until the infants were at least six months of age (Online Resource 1, Table S1). Participants were chosen at random and due to time constraints only three pairs were included (see Online Resource 1 for more on the study design). Two of the dyads lived in Canada (MOM-CHIL 1 and 3), and the other in Western Europe (MOM-CHIL 2). Participants were asked to compete dietary and health surveys for themselves and their infant (see summaries in Online Resource 1 and survey example in Online Resource 2). All three dyads reported an omnivorous diet. All mothers exclusively breastfed for ~ 4 months. MOM 1 breastfed exclusively until her child was 4 months of age, when formula was introduced. Solid foods were introduced at 6 months of age and a reduction in breastfeeding occurred between 6 and 9 months, after which it ceased. The child consumed formula until 12 months of age. MOM 2 similarly breastfed exclusively until her child was 4 months, when solids were introduced, with the gradual removal of breastmilk from the infant’s diet occurring between 9 and 12 months. MOM 3 was not able to breastfeed exclusively from birth (for explanation see Online Resource 1) so until 8 weeks of age her child consumed a mix of formula and breastmilk. However, after 8 weeks it became possible for MOM 3 to exclusively breastfeed, which she continued until the child was 4 months, when solids were introduced. Breastmilk became less important in the child’s diet at 7 months and weaning ended at 12 months.

### CSIA-AA

Debris from fingernail samples was meticulously removed using a scalpel. After cleaning, the samples were degreased following the protocol established by Tea and colleagues (2013), involving an ethyl acetate wash followed by a hexane wash. The samples were dried and precisely weighed (6 mg). In preparation for CSIA-AA, a combination of protocols from Corr et al. (2007a, b); Styring et al. (2010); and Schwartz-Narbonne et al. (2015) were followed (see Online Resource 1 for details). For analysis, samples were dissolved in ethyl acetate (1 mL) and injected twice into the Thermo Trace GC 1310 gas chromatograph coupled to a Thermo Scientific Delta V Advantage IRMS via a GC Iso-Link II Combustion interface. The measurements are reported in per mil (‰) and calibrated to VPDB.

The *δ*^13^C values reported here are a mean of duplicate *δ*^13^C measurements. Amino acid *δ*^13^C measurements were adjusted using specific correction factors to account for additional carbon added during derivatization and for the kinetic isotope effect. The correction factors were calculated using Docherty et al.’s (2001) equation (presented in Online Resource 1, equation S1). As correction factors introduce an additional source of error, the total error generated for each AA was calculated using equation S2 in Online Resource 1, as presented by Docherty et al. (2001) and Soncin et al. (2021). Additionally, a standard AA mixture of alanine, valine, proline, glutamate, and phenylalanine (purchased through Sigma-Aldrich, UK, derivatized in-house) was analyzed after every 3 to 6 sample injections, and the averages from the standard mixture were used for the calibration of the samples. True *δ*^13^C of standards were measured by EA-IRMS: Val, − 32.2 ± 0.2‰; Glu, − 32.0 ± 0.4‰; Ala, − 29.5 ± 0.0‰; Phe, − 21.3 ± 0.6‰; Pro, − 22.8 ± 0.5‰; Gly, − 33.7 ± 0.1‰; Leu, − 34.4 ± 0.7‰; Thr, − 28.2 ± 0.6‰. The precision of AA* δ*^13^C using the Trace GC was < 0.4‰ for standards run 3-6 times per sequence throughout the analytical runs (*n* = 45), with a standard deviation of ± 0.2‰ for sample duplicates. 

## Results

Stable carbon isotope profiles were generated for all participants using CSIA data. Below, we present the *δ*^13^C profiles of glutamate and glycine to illustrate both dietary and non-dietary changes experienced by the participants. Profiles and the *δ*^13^C of all 8 AAs for each participant can be found in Online Resource 1 (Fig. S2–S7, Table S2), along with the assessment of sample molecular preservation.

### Breastfeeding and weaning – *δ*^13^C of glycine and glutamate

#### MOM-CHIL 1

**Fig. 1:**
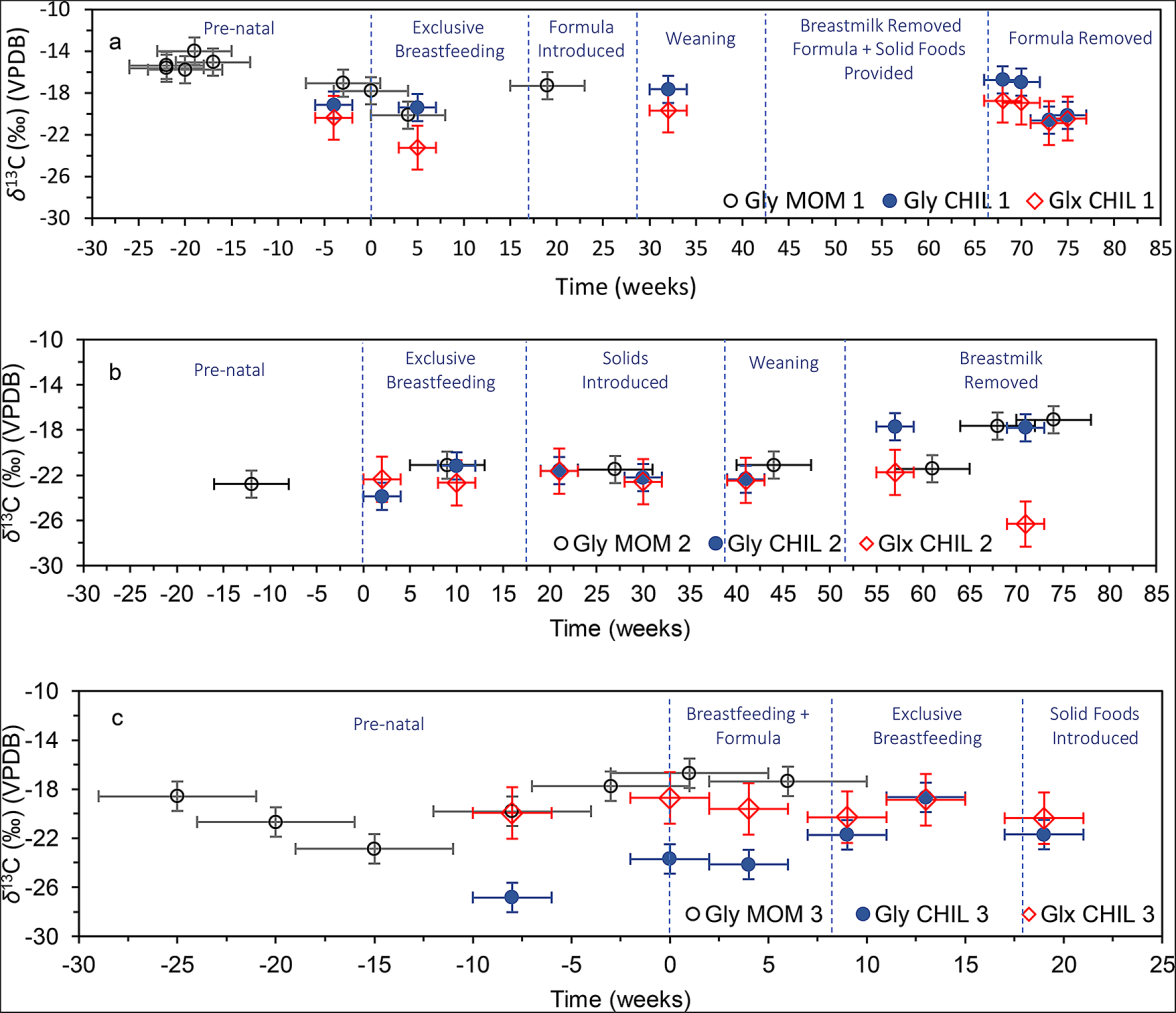
Stable carbon isotope profile for (**a**) Gly and Glx of CHIL 1 and Gly of MOM 1, (**b**) Gly and Glx of CHIL 2 and Gly of MOM 2 and (**c**) Gly and Glx of CHIL 3 and Gly of MOM 3. Zero represents birth. Error margins are 4 weeks for mothers, 2 weeks for infants, with *δ*¹³C_Gly_ and *δ*¹³C_Glx_ analytical errors of ± 1‰ and ± 2‰, respectively

MOM and CHIL 1’s *δ*^13^C_Gly_ were comparable during exclusive breastfeeding (Fig. 1a). For instance, at around 4–5 weeks, *δ*^13^C_Gly_ for the mother and infant were − 20.1‰ and − 19.4‰, respectively. Similarly, from the introduction of formula at 4.4 months until midway through weaning at 7.4 months, their *δ*^13^C_Gly_ values remained close, at − 17.3‰ and − 17.7‰, respectively.

#### MOM-CHIL 2

Figure 1b indicates that CHIL 2’s *δ*^13^C_Gly_ increased by 2.7‰ from 2 to 10 weeks, coinciding with exclusive breastfeeding. This increase aligns with the 1‰ rise observed in bulk tissue *δ*^13^C for breastfeeding infants (Fuller et al. 2003, 2006; Richards et al. 2002). Maternal *δ*^13^C_Gly_ also rose by 1.3‰ from 12 weeks before birth to 9 weeks after birth, after which it became stable until 61 weeks (1.2 years). The *δ*^13^C_Gly_ of mother and child were equivalent at ~ 9-10 weeks (–21.1‰ and − 21.2‰). Between 10 and 41 weeks (9.4 months), CHIL 2’s *δ*^13^C_Gly_ remained stable, resembling maternal isotopic compositions, but increased by 4.7‰ at 57 weeks (1.1 years). At 9 months, CHIL 2 was consuming solid foods, gradually reducing breast milk intake. The elevated *δ*^13^C_Gly_ compared to maternal isotopic compositions during this period may indicate a shift in glycine sourcing from breast milk to a post-weaning diet with a different *δ*^13^C composition. However, as the diet between the mother and infant were largely the same during the weaning and post-weaning periods (Online Resource 1), the increase in *δ*^13^C_Gly_ could also reflect a transition in the metabolic pathway for glycine from dietary routing to de novo synthesis. CHIL 2’s *δ*^13^C_Glx_ remained stable until 41 weeks (9.4 months), then declined by 4.6‰ with the reduction of breast milk intake.

#### MOM-CHIL 3

MOM and CHIL 3 exhibited a significant difference in *δ*^13^C_Gly_, ranging from ~ 4-7‰, compared to a difference of 1-2‰ in the other MOM-CHIL pairs (Fig. 1c). This substantial disparity might be due to the multiple health challenges faced by MOM 3 during her pregnancy (as detailed below) and the peripartum complications experienced by CHIL 3. For CHIL 3, *δ*^13^C_Gly_ increased by 3.1‰ from 8 weeks before birth to the week of birth (Fig. 1c). This increase may be linked to the health issues MOM 3 encountered during pregnancy, which resulted in an emergency C-section 3 weeks early. At birth, CHIL 3 experienced hypoglycemia, labored breathing, and sepsis. Initially, formula was used to supplement breastmilk until ~ 8 weeks, after which MOM 3 began exclusive breastfeeding. As a result, *δ*^13^C_Gly_ remained stable until 4 weeks due to the heavy reliance on formula, then increased by 5.5‰ until 12 weeks (3 months) with the shift to exclusive breastfeeding. Solid foods were introduced around 4 months, correlating with a 1.5‰ decrease in *δ*^13^C_Gly_ by 19 weeks (4.4 months). Fluctuations in *δ*^13^C_Glx_ were more subtle during this period; *δ*^13^C_Glx_ increased by 1.4‰ between 9 and 13 weeks (3 months) and then decreased by 1.5‰ at 19 weeks (4.4 months).

### Non-dietary changes – *δ*^13^C of glycine and glutamate

**Fig. 2:**
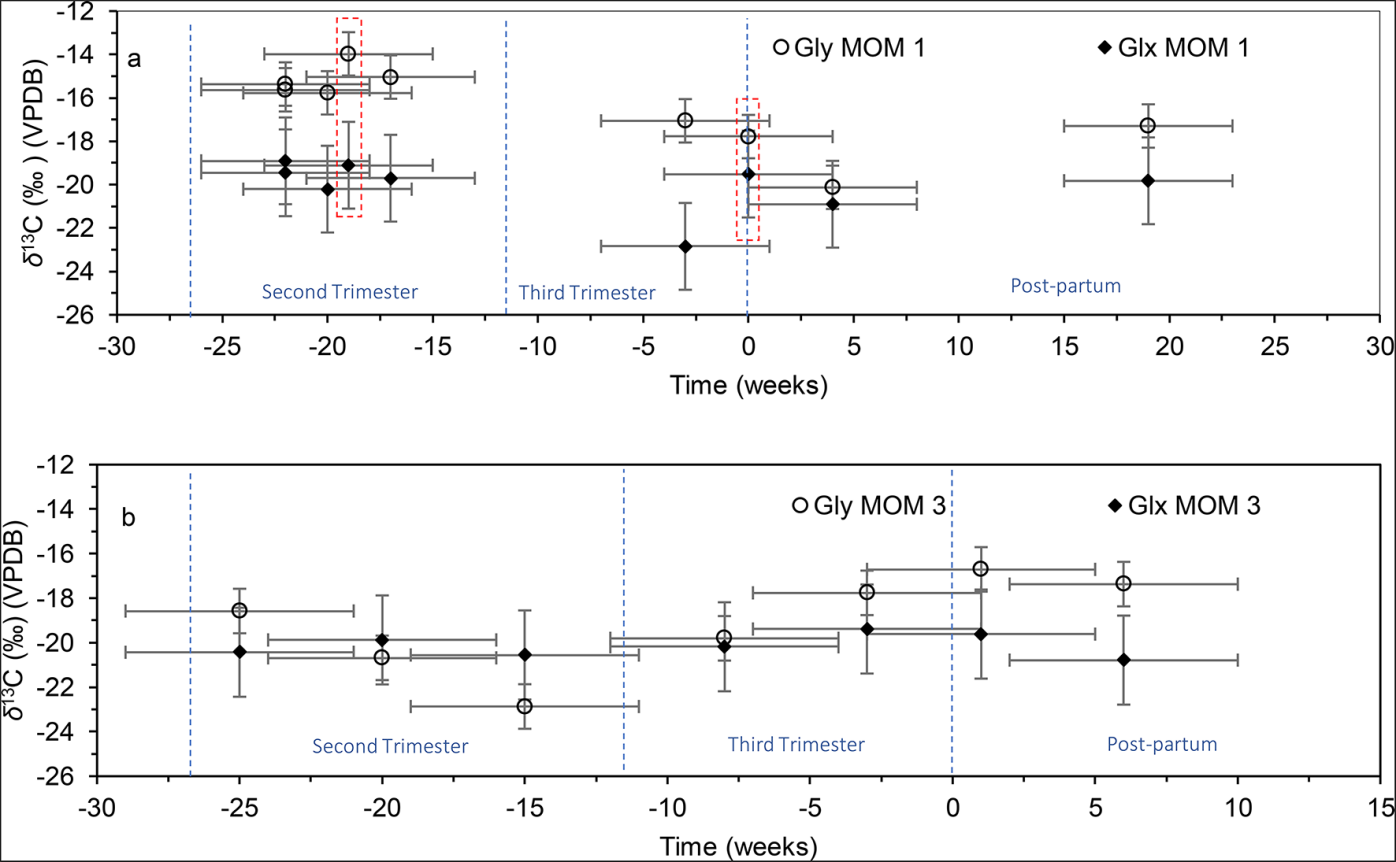
Gly and Glx *δ*^13^C for (**a**) MOM 1 from 22 weeks prior to and 19 weeks after delivery (red boxes mark the periods of stress), and (**b**) MOM 3 from 25 weeks prior to and 6 weeks after delivery. Zero represents the week of delivery. The error margin for the time period represented by each sample is 4 weeks with *δ*¹³C_Gly_ and *δ*¹³C_Glx_ analytical errors of ± 1‰ and ± 2‰, respectively

In addition to breastfeeding and weaning, glycine and glutamate *δ*^13^C profiles were effective in identifying non-dietary factors for the mothers. For instance, in Fig. 2a, MOM 1’s *δ*^13^C for glycine and glutamate increased by 1.8‰ and 1.1‰, respectively, over a single week (20 to 19 weeks before birth), corresponding to the exact period MOM 1 had COVID-19. Over the 3rd trimester, glycine and glutamate *δ*^13^C declined by 2.0‰ and 3.2‰, respectively. However, with only two data points at the start and end of the 3rd trimester, it is difficult to draw definitive conclusions for this period. Notably, between 3 weeks before and the week of delivery, *δ*^13^C_Glx_ increased by 3.3‰, likely due to the catabolic effects of late term pregnancy and giving birth, similar to the increase observed during COVID-19. This increase was not observed in *δ*^13^C_Gly_.

Figure 2b presents the glycine and glutamate *δ*^13^C profile of MOM 3 from 25 weeks prior to and 6 weeks after delivery. MOM 3’s δ^13^C_Gly_ declined by 4.3‰ from 25 to 15 weeks prior to delivery (during the second trimester). During this period, MOM 3 experienced leg and feet edema, increased migraines, and fatigue. Between 15 weeks before and 1 week after delivery, MOM 3’s *δ*^13^C_Gly_ rose by 6.2‰. In the third trimester, MOM 3 faced multiple health issues, including respiratory syncytial virus (RSV), which progressed to pulmonary pneumonia. She also experienced preeclampsia, severe anemia, and an ear infection. Due to preeclampsia complications, MOM 3 was induced 3 weeks before her due date. In contrast, *δ*^13^C_Glx_ for MOM 3 was stable until 8 weeks prior to delivery, at which point it rose by only 0.8‰. Between 3 weeks before and 6 weeks after delivery, *δ*^13^C_Glx_ steadily declined by 1.4‰. Postpartum, MOM 3 underwent various medical treatments, including two different blood pressure medications, iron supplements, antibiotics for the ear infection, a puffer to alleviate pneumonia, and pain medications due to an emergency C-section.

### Overall diet of mother-infant dyads

EAAs like phenylalanine, unlike NEAAs, are reliable diet composition indicators (Jim et al. 2006; Hare et al. 1991; Howland et al. 2003; McMahon et al. 2010). Table S3 in Online Resource 1 shows the average *δ*^13^C_Phe_ and *δ*^13^C_bulk_ of keratin samples. Study participants’ average *δ*^13^C_Phe_ ranged from − 26.1 to − 23.3‰, averaging − 24.4 ± 1.3‰. MOM and CHIL 2’s *δ*^13^C for bulk tissue and phenylalanine was 2–3‰ lower compared to the other pairs, likely due to their residence in Western Europe during pregnancy and nursing, in contrast to the other pairs, who were in Canada. Despite thier supermarkets offering similar food items, North Americans and Europeans exhibit different tissue carbon isotopes, largely due to variations in livestock feed, particularly corn (Bataille et al. 2020; Nardoto et al. 2006; Schmidt et al. 2005), and high levels of corn-derived sugars in processed foods in North America (Jahren et al. 2006). Alternatively, specific dietary choices, like MOM 2 avoiding processed foods and soft drinks, may also play a role.

As mentioned, researchers have used Δ^13^C_Gly−Phe_ to distinguish terrestrial and aquatic-based diets (Corr et al. 2005; Honch et al. 2012; Webb et al. 2018). Here, we found wide intra-individual Δ^13^C_Gly−Phe_ variability of − 3.0 to 10.6‰ (Fig. S8 in Online Resource 1). For example, MOM 1 exhibited Δ^13^C_Gly−Phe_ values ranging from 3.4 to 10.6‰, while MOM 2 ranged from 3.4 to 9.6‰. Despite these values, dietary and health surveys indicate that participants consumed a C_3_-dominant, omnivorous diet with minimal intake of aquatic food. Additionally, we noted that infants generally exhibited lower Δ^13^C_Gly−Phe_ compared to their mothers (i.e. MOM 3 avg. 4.3 vs. CHIL 3 avg. 0.6).

## Discussion

### Breastfeeding and weaning

Our study demonstrates that *δ*^13^C_Gly_ effectively tracks breastfeeding and weaning practices. CHIL 1 and 2’s profiles (Fig. 1a-b) illustrate glycine’s role in detecting the onset of exclusive breastfeeding. Specifically, during exclusive breastfeeding, *δ*^13^C_Gly_ for CHIL 1 and 2 (averaging − 18.5‰ and − 22.2‰) closely matched those of their mothers (–18.7‰ and − 21.2‰), indicating maternal dietary influence. Postpartum, an increase in MOM 2’s *δ*^13^C_Gly_ corresponded to a 2.7‰ rise in CHIL 2’s *δ*^13^C_Gly_ between 2 and 10 weeks, while the other AAs remained stable. However, from 9 to 13 months, CHIL 2’s *δ*^13^C_Gly_ increased significantly by 4.7‰, diverging from MOM 2’s stable *δ*^13^C_Gly_. More specifically, at 9 months CHIL 2’s diet comprised half breastmilk and half solid foods, and *δ*^13^C_Gly_ mirrored maternal values, but by 13 months, breastmilk was completely removed from the diet and CHIL 2’s *δ*^13^C_Gly_ (–17.7‰) became distinct from the mother’s (–21.4‰). Substantial changes in *δ*^13^C_Gly_ after the removal of breastmilk suggests that either this AA began to reflect a post-weaning diet with a different isotopic composition compared to the mother’s diet, or it began to be biosynthesized. The former scenario is plausible as CHIL 2’s *δ*^13^C_Gly_ becomes more akin to reported values for a C_3_ diet consisting of soybean meal, barley, and alfalfa (–17.3‰) (Hare et al. 1991), or durum wheat (ranging from − 17.3 to − 15.0‰) (Paolini et al. 2015). However, given that the mother and infant’s diets were largely similar, it is more likely that the observed change is due to de novo synthesis.

Unlike CHIL 2, the data for MOM-CHIL 3 present a distinct scenario. The difference in *δ*^13^C_Gly_ between MOM 3 and CHIL 3 range from 4 to 7‰, notably higher than other pairs in our study. While one interpretation could be endogenous glycine synthesis in the infant, the *δ*^13^C_Gly_ profile suggests an alternative explanation. From the week of birth to 4 weeks after, the *δ*^13^C of phenylalanine, glycine, and glutamate for CHIL 3 remained relatively stable (see Fig. 2b, and Fig. S7 in Online Resource 1), indicating that the overall diet was uniform during the first few weeks of life. This aligns with the fact that CHIL 3 was in neonatal intensive care unit (NICU) and was fed formula alongside breastmilk until ~ 8 weeks. By 13 weeks a 5.5‰ increase in *δ*^13^C_Gly_ occurred as CHIL 3 was now breastfed exclusively. Additionally, *δ*^13^C_Gly_ became depleted at 19 weeks after birth (by 2.8‰), at approximately the age CHIL 3 was introduced to solid foods. This pattern affirms that glycine was incorporated and influenced by breastmilk consumption. Consequently, we propose that the disparity in *δ*^13^C_Gly_ between CHIL 3 and MOM 3 arises from a change in the biosynthetic pathway of glycine induced by pathological stress (see below).

Glutamate *δ*^13^C also provided insights into dietary changes for infants. For example, CHIL 2’s *δ*^13^C_Glx_ decreased by 4.6‰ after the cessation of breastmilk consumption. Similarly, CHIL 3’s *δ*^13^C_Glx_ increased by 1.4‰ during exclusive breastfeeding and then decreased by 1.5‰, 19 weeks after birth, following the introduction of solid foods. The subtle changes in *δ*^13^C_Glx_ in comparison to *δ*^13^C_Gly_ in CHIL 3 were surprising as NEAAs are expected to demonstrate a greater trophic level effect than EAAs. However, as the biosynthesis of NEAAs requires a large amount of energy, under conditions of high NEAA concentrations in the diet, these AAs can be routed from dietary sources (Ambrose and Norr 1993; Newsome et al. 2014). Considering that breastmilk contains high glutamate concentrations, infants may have directly sourced glutamate from their diet during certain periods (Davis et al. 1994). The combined metabolic pathways (direct routing and biosynthesis) of glutamate may have led to variable changes in *δ*^13^C_Glx_ during infancy. In contrast, the distinctive CEAA role of glycine in infants resulted in patterns closely mirroring those of their mothers during exclusive breastfeeding, notably evidenced by the postpartum increases in *δ*^13^C_Gly_. The postpartum increase in maternal *δ*^13^C_Gly_ may be associated with the physiological/caloric burden of breastfeeding as nutritional stress can lead to elevated *δ*^13^C (further explained below).

### AA biosynthetic pathways and stress

Before delving into how pathological or physiological conditions can change the *δ*^13^C of AAs, it is crucial to grasp the intricacies of AA biosynthetic pathways. Typically, NEAAs are synthesized during glycolysis, a process where glycolytic precursors (i.e., glucose arising from carbohydrates) are transformed to form lipids. Carbon in glycine is directly linked to carbohydrate digestion, while glutamic acid is synthesized from the pool of carbon from all macronutrients including protein (Soncin et al. 2021). Various stressors, whether nutritional, physiological, or pathological, can induce a reversal in metabolic pathways, prompting the breakdown of glycogen (the stored form of carbohydrates) and lipids to produce glucose for energy. This process, known as gluconeogenesis, involves reactions with NEAAs from glycogen, leaving the remaining AAs in the body’s pool, not involved in gluconeogenesis, enriched in ^13^C (Kaleta et al. 2011; Newsome et al. 2014). In addition to glycolysis and gluconeogenesis, normal mammalian metabolism includes extensive turnover and degradation of AAs (Adeniyi-Jones et al. 1981; Berg et al. 2012). Most AA catabolism occurs through transamination, a chemical reaction transferring an amino group from one AA (donor) to a ketoacid (acceptor) to form new AAs, influencing *δ*^15^N (McMahon and McCarthy 2016). Conversely, decarboxylation, which removes a carboxyl group and releases carbon dioxide, is associated with significant effects on *δ*^13^C (Fry and Carter 2019). This process enriches the residual AA pool in ^13^C, leading to the enrichment of ^13^C in keratin formed from this pool (DeNiro and Epstein 1977).

Two mothers in our study experienced physiological or pathological stress. MOM 1, who had COVID-19, showed elevated *δ*^13^C for glycine and glutamate, possibly due to catabolic breakdown. It should be noted that during this period, MOM 1 did not report any dietary changes. MOM 3 saw a significant decline in *δ*^13^C_Gly_ (by 4.3‰) during her second trimester, possibly linked to increased AA synthesis or glycolysis from maternal and fetal growth. In her third trimester, MOM 3’s *δ*^13^C_Gly_ increased substantially (by 5.1‰) alongside multiple health issues. These trends suggest that breastfeeding-related stress could also elevate postpartum *δ*^13^C_Gly_ in mothers and subsequently in infants, given glycine’s role as a CEAA during infancy, allowing researchers to trace breastfeeding patterns.

These interpretations are supported by Fry and Carter’s (2019) investigation of *δ*^13^C of carboxyl groups (*δ*^13^C_CARBOXYL_) in AAs of keratin samples from humpback whales. Their study proposes that AA uptake from diet and new AA synthesis lower *δ*^13^C_CARBOXYL_ while catabolic effects (gluconeogenesis or decarboxylation) increase *δ*^13^C_CARBOXYL_. Carboxyl carbon represents one quarter of the carbon comprising an AA and is related to CSIA results that measure the average isotopic composition for non-carboxyl carbon (NCC) as well as carboxyl carbon. Fry and Carter’s (2019) results for humpback whales demonstrate ^13^C-enrichment in *δ*^13^C_CARBOXYL_ of both EAAs and NEAAs relative to bulk tissue *δ*^13^C due to extended fasting (> 9 months). Fry and Carter (2019) further note a greater ^13^C-enrichment in EAAs than NEAAs. They suggest that these processes may exert a more pronounced influence on EAAs, as they are not replenished from the diet during fasting, leading to a decreasing supply as they become catabolized. In contrast, NEAAs are continuously synthesized, representing a partially replenished reservoir, and consequently exhibiting less overall ^13^C enrichment than EAAs (Fry and Carter 2019).

With this is mind, the substantial difference between mother and infant *δ*^13^C_Gly_ (4–7‰) in MOM-CHIL 3 is most likely caused by the combined effects of gluconeogenesis, catabolism, and increasing demand for AA synthesis. CHIL 3’s *in utero δ*^13^C_Gly_ (–26.8‰) was the most ^13^C depleted compared to all other AAs for the mother and child, except for valine (mother’s *δ*^13^C_Val_: − 26.8‰; child’s *δ*^13^C_Val_ : − 28.9‰). Although CHIL 3’s *δ*^13^C_Gly_ eventually rose over time in response to breastmilk consumption, values remained depleted of ^13^C in comparison with the mother’s *δ*^13^C_Gly_. Given the health complications of MOM 3 during pregnancy and CHIL 3 in the first 6 months of life, it is conceivable that, in addition to insufficient body reserves, new AA synthesis, and pathological stress, glycine metabolic pathways may have been altered to support the growing infant’s needs.

In another study, Zignego et al. (2015) investigated human chondrocytes’ response to moderate compression. Based on changes in the concentrations of threonine, homoserine, and allothreonine, Zignego et al. (2015) suggested that rates of glycine, serine, and threonine metabolism were increased after mechanical loading. Similarly, Mickiewicz et al.’s (2015) analysis of metabolites in synovial fluid indicated that an anterior cruciate ligament reconstruction injury resulted in altered pathways for the metabolism firstly of glycine, serine, and threonine, then other AAs such as proline, alanine and glutamate.

Based on these studies, we can conclude that unexpected patterns may be observed in some AAs, such as glycine. We propose that as carbon for glycine is linked to carbohydrate digestion, any factors affecting glycolysis would directly affect *δ*^13^C_Gly_. During periods of stress, such as extended fasting, glycogen, the stored form of glucose, is the first to be broken down by the body to obtain energy, followed by lipids and protein (Fry and Carter 2019). As such, *δ*^13^C_Gly_ would be one of the first AAs to be changed by these processes. In contrast, since glutamic acid is synthesized from all macronutrients, changes in *δ*^13^C_Glx_ may be less pronounced unless lipids and proteins are also being broken down. Further research is needed to understand why other AAs involved in carbohydrate metabolism, such as alanine or serine, do not show similar significant changes.

### Overall diet

Phenylalanine is known for its minimal enrichment (1–2‰) from diet to tissue (Corr et al. 2005), making it a reliable indicator of the isotopic composition of terrestrial or marine plants at the base of the food web (e.g., Choy et al. 2010; Larsen et al. 2013; McMahon et al. 2010). The average *δ*^13^C_Phe_ for MOM 1, 2, and 3 (–24.0‰, − 26.1‰, and − 23.4‰, respectively) suggest a diet primarily composed of C_3_ plants and terrestrial livestock fed on a C_3_ diet (McCullagh et al. 2005) (Table S3). This observation is supported by the bulk *δ*^13^C averages (–19.1‰, − 21.4‰, and − 18.7‰, respectively), which also indicate a C_3_ plant-dominated diet (Burt and Amin 2014). Human collagen *δ*^13^C typically exhibits a positive offset of 3–5‰ from diet (Ambrose and Norr 1993). Subtracting 5‰ from the *δ*^13^C_bulk_ averages approximates the *δ*^13^C_Phe_, providing further evidence of limited enrichment in *δ*^13^C of phenylalanine.

#### Application of Δ^13^C_Gly−Phe_

Based on our findings, Δ^13^C_Gly-Phe_ proved unreliable for distinguishing diets heavily reliant on terrestrial versus aquatic resources due to significant intra-individual variability among participants. This issue was also noted by Choy et al. (2010) for Korean archaeological sites. Their study found that Δ^13^C_Gly-Phe_ was not definitive without considering threonine *δ*^13^C, as threonine, an EAA, exhibited distinct *δ*^13^C between marine and terrestrial protein consumers (Choy et al. 2010). Their study further highlighted the absence of a universal Δ^13^C_Gly-Phe_ threshold for distinguishing C_3_, C_4_, or marine diets. Choy et al. (2010) examined bone samples from humans (*n* = 9) and animals (*n* = 27), and while the ages and sex of most samples were not provided, one human sample was noted as coming from a 7.5-month-old; this infant exhibited the smallest Δ^13^C_Gly-Phe_ among the human samples. Similarly, Cheung et al. (2022), studying weaning practices at two Middle Neolithic communities in the Paris Basin region, Balloy and Vignely, found difficulty in interpreting Δ^13^C_Gly-Phe_, particularly in cases where individuals consumed significant freshwater protein. They suggested that small Δ^13^C_Gly-Phe_ values could reflect unusually low baseline *δ*^13^C_Gly_ in the rivers or exploitation of lower trophic resources. Building on these studies, we propose an alternative explanation based on our *δ*^13^C_Gly_ results: variability in glycine biosynthesis influenced by physiological factors, rather than baseline conditions or specific dietary choices, may play a significant role in Δ^13^C_Gly-Phe _variability.

Like Choy et al. (2010), our results indicated that infants had smaller Δ^13^C_Gly−Phe_ compared to their mothers. Cheung et al. (2022) also observed that Δ^13^C_Gly−Phe_ were smaller for most infants (< 3 years, *n* = 6) compared to the adults (*n* = 4). In one group from Vignely in France, there were no overlapping Δ^13^C_Gly−Phe_ values between adults (*n* = 2, range 15.2 to 15.7‰) and infants (*n* = 4, range 10.2 to 13.8‰). To explain why adults had larger Δ^13^C_Gly−Phe_ than the nonadults, Cheung et al. (2022) proposed that foodstuffs with lower *δ*^13^C_Gly_ and *δ*^13^C_Phe_ were consumed by both adults and infants, with the latter consuming a higher portion of such foods. Our data do not support the idea that children consumed greater quantities of lower trophic level food or foods with lower *δ*^13^C_Gly_ and *δ*^13^C_Phe_ in comparison to mothers, given that smaller Δ^13^C_Gly−Phe_ in children were also present during the prenatal period and exclusive breastfeeding, when they relied heavily on their mother’s diet. In Cheung et al.’s (2022) study, the age range of Vignely infants spanned from 0 to 2.8 years, encompassing both the fetal stage and exclusive breastfeeding period as determined through their Bayesian statistical analysis (Tsutaya 2019). Therefore, we propose that metabolic processing of glycine during fetal and infant stages primarily contributed to the observed smaller Δ^13^C_Gly−Phe_.

## Conclusions

This study utilized CSIA-AA to reconstruct breastfeeding and weaning practices. Our findings highlight that *δ*^13^C of glycine, and to a lesser extent glutamate, effectively trace the onset of exclusive breastfeeding and the cessation of weaning. The pronounced fluctuations in *δ*^13^C_Gly_, compared to other AAs, reflect glycine’s unique role as a CEAA, sourced primarily from the diet during infancy and biosynthesized later in life. Elevated *δ*^13^C_Gly_ in infants, influenced by mothers’ *δ*^13^C_Gly_ during breastfeeding, facilitated tracking of breastmilk consumption. Meanwhile, more subtle changes in *δ*^13^C_Glx_ can be attributed to glutamate’s status as a NEAA, capable of switching between biosynthesis and dietary uptake, particularly evident during exclusive breastfeeding.

Stable carbon isotope compositions of glycine and glutamate also serve as indicators of physiological and pathological stress for mothers. Glycine’s carbon is linked to carbohydrate digestion, while glutamic acid synthesis utilizes carbon from all macronutrients (carbohydrates, lipids, and proteins). Under stress, initial breakdown of glycogen affects glycine synthesis, with subsequent impacts on glutamic acid synthesis as lipid and protein breakdown occurs over longer periods. These catabolic stressors enrich these AAs in ^13^C, increasing their *δ*^13^C values.

Our study emphasizes the complexities of AA metabolic routing and its implications for dietary investigations using CSIA-AA data. The application of Δ^13^C_Gly-Phe_ to distinguish terrestrial from aquatic diets may lead to misleading interpretations due to glycine’s CEAA functionality during infancy and the influence of anabolic or catabolic effects on *δ*^13^C_Gly_ (resulting in lower or higher values). Given our current limited understanding of AA metabolic mechanisms and their relationship to *δ*^13^C, future CSIA-AA research should explore these complexities more comprehensively. Long-term studies from childhood to adulthood are crucial to elucidate the transition from CEAA to NEAA status for glycine, thereby enhancing the interpretation of stable carbon isotope data for AAs in dietary reconstruction and assessing physiological and pathological stressors effectively.

## Electronic supplementary material

Below is the link to the electronic supplementary material.

Supplementary Material 1

Supplementary Material 2

## Data Availability

Data is provided within the manuscript and the supplementary information files.
