# Supplementary material for: Amino acid stable carbon isotopes in nail keratin illuminate breastfeeding and weaning practices of mother – infant dyads
Source: Amino Acids. 2025 Jan 30;57:13. doi: 10.1007/s00726-024-03425-2 (PMC11782432; doi:10.1007/s00726-024-03425-2)
Supplement: Supplementary file 2 [file 726_2024_3425_MOESM2_ESM.docx]

**Title: Amino Acid Stable Carbon Isotopes in Nail Keratin Illuminate Breastfeeding and Weaning Practices of Mother – Infant Dyads**

Journal Name: Amino Acids

Authors: *Hana Salahuddin^1^, Andrea L. Waters-Rist^1^ ([0000-0003-1807-3171](about:blank)), Fred J. Longstaffe^2^ ([0000-0003-4103-4808](about:blank))

^1^Anthropology, Western University, London, Canada, ^2^Earth Sciences, Western University, London, Canada

*Corresponding Author: hsalahu@uwo.ca

**Online Resource 1**

Study Design

Description: Details the study approach and participant recruitment.

As this study forms part of a larger PhD project, the sample size was limited to three mother-infant dyads due to several practical constraints. Participant recruitment occurred during the COVID-19 pandemic, which significantly delayed access to laboratory facilities and restricted our ability to put out a broad call for participants. Given these time restraints and considerations of feasibility, we focused on three dyads.

The mothers were recruited based on their willingness to breastfeed for part of their child’s infancy, as this fit the primary goal of the study, which was to investigate the shifts in isotope signatures linked to breastfeeding. No other restrictions or expectations were placed on the participants. This approach was intentional, as it allowed us to observe a range of breastfeeding and weaning practices, closely reflecting real-life diversity. The only other requirement was that participants could provide at least one baseline sample before giving birth, enabling us to have comparative values for the mothers.

Although we considered including formula-fed infants as a control group, practical constraints related to time and funding prevented this. The amino acid composition of different formulas was not analyzed in this study. Since formula consumption does not cause the *δ*^13^C trophic level effect seen with breastfeeding (Fuller et al. 2006), and analyses of 10 formula varieties showed that all had lower *δ*^13^C signatures than breastmilk (Kootker et al. 2024), formula was treated similarly to other weaning foods. However, future studies should determine the amino acid *δ*^13^C signatures of formula to provide useful contextual data.

Health and Diet Survey Summaries

Description: Below are summaries of the dietary surveys completed by the three participating mothers.

MOM 1

Age: 31

Height: 156.0 cm

Weight before pregnancy: 81.6 kg

Weight at 1^st^ trimester: 81.6 kg

Weight at 2^nd^ trimester:86.2 kg

Weight at 3^rd^ trimester: 90.7 kg

Weight after pregnancy: 81.6 kg

Periods of Illness:

23^rd^ of Pregnancy: contracted COVID-19. Had persistent shortness of breath until delivery.

Dietary Information:

Consumed an omnivorous diet. Ate much more salad and vegetables postpartum than pre-partum. Experienced increased appetite during the 1^st^ trimester and loss of appetite from 2^nd^ trimester onwards.

Foods eaten often (1-3 times a week)

- meat (i.e. chicken, beef; excl. fish)
- fruits (e.g. apple, banana, orange, grape, berries, melons, lemon/lime, avocado; includes fruit juices, excludes pineapple)
- other vegetables (e.g., peppers, tomato, celery, broccoli, cauliflower, garlic, cucumber, mushrooms)
- diary (e.g. milk, yogurt, cheese)
- eggs
- grains (e.g. rice, bread, couscous, quinoa, pasta)

Foods eaten occasionally (once a month)

- fish or shellfish
- pork
- beans (e.g. tofu, chickpeas, green, kidney, black, lima, string beans)
- root vegetables (e.g. onion, beet, carrot, potato)
- leaf and leaf stalk vegetables (e.g. lettuce, cabbage, spinach, brussels sprouts)

CHIL 1

Height at the time of birth: 50.0 cm

Height at 6 months: 73.0 cm

Weight the time of birth: 3.9 kg

Weight at 6 months: 8.6 kg

Periods of Illness:

None.

Dietary Information:

4 Months: exclusively breastfed up till this point. Introduced formula, ~ 3-4 times a day.

6 Months: began weaning and completed weaning by 9 months.

9 Months: began to only consume formula and certain solid foods.

Foods consumed often during weaning (1-3 times a week)

- fruits (e.g. apple, banana, orange, grape, berries, melons, lemon/lime, avocado; includes fruit juices, excludes pineapple)
- root vegetables (e.g. onion, beet, carrot, potato)
- grains (e.g. rice, bread, couscous, quinoa, pasta)

Foods consumed often post-weaning (1-3 times a week)

- fruits (e.g. apple, banana, orange, grape, berries, melons, lemon/lime, avocado; includes fruit juices, excludes pineapple)
- root vegetables (e.g. onion, beet, carrot, potato)
- grains (e.g. rice, bread, couscous, quinoa, pasta)

MOM 2

Age: 35

Height: 164.7 cm

Weight before pregnancy: 68.0 kg

Weight at 1^st^ trimester: n/a

Weight at 2^nd^ trimester: n/a

Weight at 3^rd^ trimester: 79.4 kg

Weight after pregnancy: 72.6 kg

Periods of Illness:

3 Months after Birth: had an ear infection and was on antibiotics (amoxycillin) to treat it.

Dietary Information:

Consumed an omnivorous diet.

Foods eaten often (1-3 times a week)

- meat (i.e. chicken, beef; excl. fish)
- fruits (e.g. apple, banana, orange, grape, berries, melons, lemon/lime, avocado; includes fruit juices, excludes pineapple)
- root vegetables (e.g. onion, beet, carrot, potato)
- leaf and leaf stalk vegetables (e.g. lettuce, cabbage, spinach, brussels sprouts)
- other vegetables (e.g., peppers, tomato, celery, broccoli, cauliflower, garlic, cucumber, mushrooms)
- olives (including olive oil)
- diary (e.g. milk, yogurt, cheese)
- grains (e.g. rice, bread, couscous, quinoa, pasta)

Foods eaten occasionally (once a month)

- pork
- beans (e.g. tofu, chickpeas, green, kidney, black, lima, string beans)
- peas
- lentils
- corn and corn-based products
- nuts and nut-based products
- eggs

Pre- and Postpartum: craved fruit (i.e. grapes, strawberries, pineapple, apple, banana, other berries, etc.). Experienced increased appetite, especially during the 3^rd^ trimester and while breastfeeding. Tried to eat healthy; no fizzy drinks and little processed food.

CHIL 2

Height at the time of birth: n/a

Height at 6 months: 68.0 cm

Height at 12 months: 72.8 cm

Weight the time of birth: 1.7 kg

Weight at 6 months: 3.2 kg

Weight at 12 months: 4.3 kg

Periods of Illness: None.

Dietary Information:

~ 4 Months: Was exclusively breastfed up to this point. Introduced solids.

6 Months: began to consume more solids; ~ ¼ solids and ¾ breastmilk. Provided formula to supplement breastmilk. However, formula consumption was limited to 1-2 bottles per week.

9 Months: 50:50 ratio between solids and breastmilk.

12 Months: stopped breastfeeding and began drinking cow’s milk, ~200-400mL per day.

Foods consumed often during weaning (1-3 times a week)

- meat (chicken and beef)
- beans (e.g. tofu, chickpeas, green, kidney, black, lima, string beans)
- fruits (e.g. apple, banana, orange, grape, berries, melons, lemon/lime, avocado; includes fruit juices, excludes pineapple)
- root vegetables (e.g. onion, beet, carrot, potato)
- leaf and leaf stalk vegetables (e.g. lettuce, cabbage, spinach, brussels sprouts)
- other vegetables (e.g., peppers, tomato, celery, broccoli, cauliflower, garlic, cucumber, mushrooms)
- olives (including olive oil)
- diary (e.g. milk, yogurt, cheese)
- foods sweetened with natural sugars (i.e. sugarcane)

Foods consumed often post-weaning (1-3 times a week)

- meat (chicken and beef)
- beans (e.g. tofu, chickpeas, green, kidney, black, lima, string beans)
- fruits (e.g. apple, banana, orange, grape, berries, melons, lemon/lime, avocado; includes fruit juices, excludes pineapple)
- root vegetables (e.g. onion, beet, carrot, potato)
- leaf and leaf stalk vegetables (e.g. lettuce, cabbage, spinach, brussels sprouts)
- other vegetables (e.g., peppers, tomato, celery, broccoli, cauliflower, garlic, cucumber, mushrooms)
- olives (including olive oil)
- diary (e.g. milk, yogurt, cheese)
- foods sweetened with natural sugars (i.e. sugarcane)

MOM 3

Age: 30
Height: 160.0 cm
Weight before pregnancy: 70.3 kg

Weight at 1^st^ trimester: 72.6 kg

Weight at 2^nd^ trimester: 79.4 kg

Weight at 3^rd^ trimester: 90.7 kg

Weight after pregnancy: 81.6 kg

Periods of Illness:

1^st^ Trimester: constant nausea, vomiting, and heartburn; medication to alleviate the symptoms was taken. The nausea, vomiting, and heartburn symptoms lasted the entire pregnancy and heartburn continued after pregnancy.

2^nd^ Trimester: developed leg and feet edema. Increase in migraines and fatigue.

3^rd^ Trimester: leg and feet edema continued. Contracted RSV, which then developed into pulmonary pneumonia. Also was severely anemic, diagnosed with preeclampsia, and had an ear infection. Due to preeclampsia complications, was induced 3 weeks prior to the due date. After giving birth, took two different blood pressure medication, as well as iron medication, antibiotics for the ear infection, a puffer to alleviate the pneumonia, and pain medications from the emergency C-section.

Dietary Information:

Overall diet was omnivorous. Consumed a low salt diet from the 2^nd^ trimester to 6 months after giving birth due to swelling in legs, feet, and hands.

Foods eaten often (1-3 times a week)

- meat (i.e. chicken, beef; excl. fish)
- beans (e.g. tofu, chickpeas, green, kidney, black, lima, string beans)
- lentils
- fruits (e.g. apple, banana, orange, grape, berries, melons, lemon/lime, avocado; includes fruit juices, excludes pineapple)
- root vegetables (e.g. onion, beet, carrot, potato)
- leaf and leaf stalk vegetables (e.g. lettuce, cabbage, spinach, brussels sprouts)
- Other vegetables (e.g., peppers, tomato, celery, broccoli, cauliflower, garlic, cucumber, mushrooms)
- Nuts and nut-based products
- Diary (e.g. milk, yogurt, cheese)
- Eggs
- Grains (e.g. rice, bread, couscous, quinoa, pasta)

Foods eaten occasionally (once a month)

- Fish or shellfish
- Pineapple

Pre- and Postpartum: avoided unpasteurized ingredients (e.g. honey, cheeses, etc.), cold cut meats, raw fish and eggs (e.g. sushi, hollandaise sauce, eggs over medium, etc.), fast food meals that involved ingredients being left out for the day (e.g. booster juice, pita pit, subway, etc.) and alcohol. Cravings in the 1^st^ trimester included cucumbers with vinegar and salt, lime, white chocolate scones, and McDonalds fries with vanilla ice cream. For the 2^nd^ and 3^rd^ trimesters, craving included crepes, WORKS burgers and meals with bread or potato. Food aversions included fish (all the time) and occasionally chicken. After pregnancy, resumed a regular diet, with a continued aversion to fish. Also avoided some unpasteurized ingredients like honey. Appetite slightly increased but due to heartburn, nausea, and vomiting, diet quantity was impacted. Meals became smaller and more frequent. After pregnancy appetite and thirst increased due to breastfeeding.

CHIL 3

Height at the time of birth: 48.3 cm

Height at 6 months: 81.3 cm

Weight the time of birth: 2.9 kg

Weight at 6 months: 8.2 kg

Periods of Illness:

After Birth: infant was in neonatal intensive care unit (NICU) for 3 days due to an episode of hypoglycemia. Had laboured breathing and was septic.

Between 2 to 8 Months: developed three respiratory illnesses.

At 4 Months: developed a lactose allergy (could not drink animal milk, e.g. cow or goat).

Dietary Information:

After Birth: was provided the Similiac Pro-Advance formula to supplement while breastmilk came in.

~ 8 Weeks: switched to exclusive breastfeeding.

4 Months: Solids introduced. Purees were given as a snack ~1-2 times a day. Purees included chicken, vegetables (corn, peas, carrots, squash, etc.) and fruit (papaya, mango, bananas, avocado, etc.).

6 Months: Water and natural juices were introduced.

7- 8 Months: Due to low breastmilk, Enfamil Lactose-Free formula was introduced. The formula was mixed breastmilk at a 1:1 ratio. Mixed bottles were given 3 times a day, approximately 2 to 3 times a week.

Fingernail Samples

Description: This section details the methods used to calculate the time represented by each fingernail sample.

Fingernails, on average, grow between 0.5 to 1.2 mm per week, with a decrease in this rate observed with age (Bean 1974; Hillman 1955; Kandil 1972). The samples we collected from mothers ranged between 2-3 mm, while those from infants measured between 0.5-1.5 mm. Since fingernails grow from the cuticle but are collected at the fingertip, there is a lag estimated to be 4-6 months for adults and 2-3 months for infants (Fogel et al. 1989; Fuller et al. 2006). Based on these growth rates, nail clippings represent a maximum of 6 months prior to the date collected for the mothers and a maximum of 3 months for the children. To calculate the time represented by each adult sample, 4-6 months (or 2-3 months for subadults) was subtracted from the date of sample collection and then a median date was determined from the aforementioned range (Table S1). The date was converted to the number of weeks before or after birth, where 0 represents the week of birth.

Table S1: Fingernail samples from participants and the time period each represents.

| **Mother /Child** | **Sample code** | **Week sample was collected (# of weeks from birth)^a^** | **Time range (weeks)** | **Median (weeks)** | **Bulk or CSIA** |
| --- | --- | --- | --- | --- | --- |
| **Pair 1** | | | | |  |
| MOM 1 | MYN1 | 0 | -26 – -18 | -22 | Both |
|  | MYN2^b^ | 0 | -26 – -18 | -22 | CSIA |
|  | MYN3 | 2 | -24 – -16 | -20 | Both |
|  | MYN4 | 4 | -23 – -15 | -19 | Both |
|  | MYN5 | 6 | -21 – -13 | -17 | Both |
|  | MYN6 | 20 | -7 – 1 | -3 | Both |
|  | MYN7 | 23 | -4 – 4 | 0 | Both |
|  | MYN8 | 26 | -0 – 8 | 4 | Both |
|  | MYN9 | 40 | 15 – 23 | 19 | Both |
| CHIL 1 | MTN1 | 7 + 10^c^ | -6 – -2 | -4 | CSIA |
|  | MTN2 | 13 + 18 | 3 – 7 | 5 | CSIA |
|  | MTN3 | 42 + 44 | 30 – 34 | 32 | CSIA |
|  | MTN4 | 79 | 66 – 70 | 68 | Both |
|  | MTN5 | 82 | 68 – 72 | 70 | Both |
|  | MTN6 | 84 | 71 – 75 | 73 | Both |
|  | MTN7 | 86 | 73 – 77 | 75 | Both |
| **Pair 2** | | | | |  |
| MOM 2 | MSN1 | 97 + 94 | 70 – 78 | 74 | Both |
|  | MSN2 | 91 + 87 | 64 – 72 | 68 | Both |
|  | MSN3 | 83 | 57 – 65 | 61 | Both |
|  | MSN4 | 67 + 64 | 40 – 48 | 44 | Both |
|  | MSN5 | 50 + 47 | 23 – 31 | 27 | Both |
|  | MSN6 | 32 + 30 | 5 – 13 | 9 | Both |
|  | MSN7 | 11 + 10 | -16 – -8 | -12 | Both |
| CHIL 2 | MLN1 | 87 + 76 | 69 – 73 | 71 | CSIA |
|  | MLN2 | 74 + 61 | 55 – 59 | 57 | CSIA |
|  | MLN3 | 55 + 48 | 39 – 43 | 41 | CSIA |
|  | MLN4 | 45 + 37 | 28 – 32 | 30 | CSIA |
|  | MLN5 | 35 + 27 | 19 – 23 | 21 | CSIA |
|  | MLN6 | 25 + 16 | 8 – 12 | 10 | CSIA |
|  | MLN7 | 14 + 10 | 0 – 4 | 2 | CSIA |
| **Pair 3** | | | | |  |
| MOM 3 | MAN1 | -3 | -29 – -21 | -25 | Both |
|  | MAN2 | 1 | -24 – -16 | -20 | Both |
|  | MAN3 | 6 | -19 – -11 | -15 | Both |
|  | MAN4 | 14 | -12 – -4 | -8 | Both |
|  | MAN5 | 18 | -7 – 1 | -3 | Both |
|  | MAN6 | 23 | -3 – 5 | 1 | Both |
|  | MAN7 | 27 | 2 – 10 | 6 | Both |
| CHIL 3 | MCN1 | 1 + 6 | -10 – -6 | -8 | Both |
|  | MCN2 | 10 | -2 – 2 | 0 | Both |
|  | MCN3 | 14 | 2 – 6 | 4 | Both |
|  | MCN4 | 18 | 7 – 11 | 9 | Both |
|  | MCN5 | 23 | 11 – 15 | 13 | Both |
|  | MCN6 | 27 | 17 – 21 | 19 | Both |

^a^ Zero represents the week of birth; negative values indicate weeks prior to delivery.

^b^ Replicate of sample MYN1.

^c^ Plus sign indicates that two samples collected at two different times were combined.

CSIA-AA Method

Description: This section details the CSIA-AA method employed to analyze the *δ*^13^C values of AAs. It also includes information on the correction factors applied to adjust *δ*^13^C values, the quality control measures implemented to ensure data accuracy.

First, the air from sample vials containing 6 mg of material was replaced with dry nitrogen (N_2_). The samples then underwent hydrolysis by heating in 6N HCl for 24 hours at 100 –110°C to break down proteins into their constituent AAs. In this step, glutamine (Gln) converts to its free-form, glutamic acid (Glu), by shedding a mineral ion, and its isotopic composition is reported as glutamate (Glx). Following the hydrolysis step, HCl was evaporated under dry N_2_ at 40 – 60°C, and samples were derivatized as *N*-acetyl methyl esters (NACME). Derivatization requires two reactions, esterification and acetylation. For esterification, an acidified methanol solution was prepared by combining 0.8 mL of acetyl chloride with 5 mL of anhydrous methanol. One mL of this solution was added to the samples. The air in the sample vials was replaced with dry N_2_ and samples were heated at 75°C for 1 hour and evaporated under dry N_2_ in an ice bath. For acetylation, a solution of acetic anhydride (1mL), triethylamine (2 mL), and dichloromethane (5 mL) was prepared and 1 mL of this solution was added to the sample. The air in the sample vials was replaced with dry N_2_ and samples were heated at 60°C for 10 minutes and evaporated under dry N_2_ in an ice bath. To eliminate insoluble and high-polarity by-products, ethyl acetate and saturated NaCl solution were added to the sample. The low-polarity ethyl acetate layer, containing the sample, was separated using a pipette and transferred to a new vial, while the undesirable by-products were discarded with the saturated NaCl solution layer. The ethyl acetate-extracted sample was then evaporated under dry N_2_ in an ice bath. The samples were stored in the freezer at -25°C until ready for analysis.

Correction Equations for *δ*^13^C Measurements of AAs

Equation S1: ${n_{\mathrm{cd}}\delta}^{13}C_{\mathrm{cd}}= n_{c}\delta^{13}C_{c}+ n_{d}\delta^{13}C_{d}$

n = number of moles of carbon

c = compound of interest

d = derivative group

cd = derivatized compound

Equation S2: $\sigma^{2}=\sigma_{S}^{2}\left( \frac{n_{S}}{n_{C}} \right)^{2}+ \sigma_{DS}^{2}\left( \frac{n_{S}+ n_{D}}{n_{C}} \right)^{2}+ \sigma_{DC}^{2}\left( \frac{n_{C}+n_{D}}{n_{C}} \right)^{2}$

σ = standard deviation

*n* = number of carbon atoms

S = nonderivatized standard

DS = derivatized standard

DC = derivatized compound

C = original compound

D = derivative group

The *δ*^13^C average correction factor values and SD for samples across the analytical sessions (*n* = 45) were as follows: Val, –39.5 ± 3.1‰; Glu, –40.2 ± 2.1‰; Ala, –40.7 ± 0.5‰; Phe, –46.9 ± 0.4‰; Pro, –42.5 ± 1.5‰; Gly, –40.1 ± 1.3‰; Leu, –39.0 ± 0.1‰; and Thr, –52.4 ± 1.5‰.

CSIA-AA Quality Control Criteria

Baseline resolution, which is essential for the determination of reliable compound-specific *δ*^13^C values, was obtained for eight AAs accounting for 52% of the carbon in keratin. The two most prominent AAs in keratin, cysteine (16%) and serine (12%), could not be isolated. We followed Mora et al. (2018, 2022) to assess the molecular preservation of nail keratin using AA percentages from individual peak areas obtained during gas chromatography by flame ionization detection (GC-FID). We compared these AA percentages with the values for modern and archaeological hair keratin from Mora et al. (2018), and modern nail keratin from O’Connell (2001) and Goldsmith (1991) (Figure 1). The AA percentages aligned, except for threonine, representing 3% of the total instead of the ~6% for nail keratin, and glycine, constituting 17% instead of ~4-8% (Goldsmith 1991; O’Connell et al. 2001). This dissimilarity may stem from different hydrolysis and derivatization techniques, as various procedures can yield lower or higher amounts of specific AAs (Silverman et al. 2022). In our study, we followed the conventional hydrolysis method, heating the samples at 110°C for 24 hours under anoxic conditions. Based on Silverman et al.’s (2022) review of primary AA loss mechanisms during common acid hydrolysis procedures, threonine experiences degradation during conventional hydrolysis. Silverman et al. (2022) suggests that shorter hydrolysis times (<20 h) can optimize threonine yields but may compromise other AAs.

Leucine could not be assessed as it was not detected on the GC-FID but appeared on the Trace GC, likely due to the higher sensitivity of the latter instrument. Considering the abundances obtained for the remaining AAs, we conclude that the keratin samples in this analysis effectively preserved their AA isotopic compositions.

Fraction of total

| Amino acids | Archaeological Hair (Mora et al. 2018) | Modern Hair (Mora et al. 2018) | Nail Keratin | Modern Nail (O'Connell et al. 2001) | Modern Nail (Goldsmith 1991) |
| --- | --- | --- | --- | --- | --- |
| Ala | 4 | 5 | 4 | 5 | 5.5 |
| Val | 9 | 6.5 | 5 | 5.5 | 4 |
| Gly | 4 | 4 | 17 | 4.7 | 8 |
| Thr | 8 | 10 | 3.5 | 6.5 | 6 |
| Pro | 11 | 10 | 7 | 6.8 | 6 |
| Glx | 18 | 19 | 15 | 12.7 | 13.5 |
| Phe | 4 | 4 | 5 | 2.4 | 2.5 |

Fig. S1 Fractions of the total (%) of AA peak areas measured in nail keratin samples (this study) and archaeological and modern hair from Mora et al. (2018), and modern nail from O’Connell et al. (2001) and Goldsmith (1991)

*δ*^13^C_AA_ Data

Description: This section presents the *δ*^13^C of AAs for all samples, the *δ*^13^C_AA_ profiles for each individual, and comparison of Δ^13^C_Gly-Phe_ values between mothers and infants.

Table S2. Stable carbon isotope compositions for all AAs.

| **Sample** | **Amino acid *δ*^13^C (‰) (VPDB)** | | | | | | | | | |
| --- | --- | --- | --- | --- | --- | --- | --- | --- | --- | --- |
|  | **Ala** | **Gly** | **Nor-Leu** | **Leu** | **Val** | **Pro** | **Thr** | **Glx** | **Phe** |  |
| MYNI | -21.37 | -15.36 | -28.14 | -27.02 | -26.89 | -16.67 | -7.59 | -19.45 | -24.66 |  |
| MYN2 | -21.42 | -15.63 | -28.29 | -27.21 | -27.38 | -16.88 | -7.36 | -18.90 | -24.46 |  |
| MYN3 | -23.26 | -15.77 | -28.18 | -27.21 | -27.53 | -17.29 | -7.61 | -20.21 | -24.47 |  |
| MYN4 | -21.75 | -13.97 | -28.48 | -27.69 | -27.31 | -17.56 | -8.20 | -19.11 | -24.56 |  |
| MYN5 | -21.88 | -15.04 | -27.63 | -26.88 | -27.23 | -17.43 | -8.36 | -19.70 | -24.48 |  |
| MYN6 | 21.40 | -17.06 | -26.94 | -25.24 | -27.03 | -16.54 | -7.67 | -22.85 | -23.41 |  |
| MYN7 | -20.98 | -17.79 | -27.20 | -24.76 | -26.17 | -16.58 | -7.77 | -19.51 | -23.15 |  |
| MYN8 | -22.97 | -20.12 | -27.10 | -25.28 | -27.20 | -16.81 | -7.65 | -20.90 | -23.49 |  |
| MYN9 | -22.15 | -17.30 | -26.94 | -25.51 | -26.94 | -16.86 | -8.20 | -19.82 | -23.16 |  |
|  |  |  |  |  |  |  |  |  |  |  |
| MTN1 | -22.29 | -19.15 | -28.62 | -27.15 | -28.14 | -17.34 | -8.99 | -20.37 | -25.06 |  |
| MTN2 | -24.50 | -19.39 | -29.75 | -27.96 | -28.09 | -17.05 | -8.73 | -23.23 | -25.49 |  |
| MTN3 | -20.61 | -17.65 | -27.14 | -23.88 | -24.50 | -15.28 | -8.53 | -19.67 | -22.06 |  |
| MTN4 | -21.38 | -16.74 | -26.89 | -24.05 | -24.66 | -15.59 | -8.44 | -18.73 | -22.15 |  |
| MTN5 | -21.62 | -16.96 | -26.75 | -24.55 | -25.87 | -15.89 | -9.08 | -18.92 | -22.69 |  |
| MTN6 | -24.34 | -20.60 | -26.97 | -25.20 | -27.18 | -16.44 | -9.50 | -20.88 | -23.13 |  |
| MTN7 | -24.06 | -20.14 | -27.32 | -24.97 | -26.84 | -16.79 | -9.41 | -20.45 | -22.73 |  |
|  |  |  |  |  |  |  |  |  |  |  |
| MSN1 | -24.29 | -17.09 | -28.18 | -29.16 | -29.41 | -19.08 | -9.07 | -20.99 | -26.62 |  |
| MSN2 | -26.01 | -17.65 | -28.98 | -30.38 | -30.50 | -20.02 | -9.98 | -22.01 | -27.22 |  |
| MSN3 | -26.78 | -21.43 | -27.34 | -27.95 | -29.64 | -19.72 | -10.90 | -22.73 | -25.09 |  |
| MSN4 | -26.18 | -21.10 | -26.63 | -27.17 | -28.90 | -18.59 | -9.61 | -21.89 | -25.01 |  |
| MSN5 | -26.35 | -21.50 | -28.18 | -28.40 | -29.88 | -20.15 | -11.08 | -23.83 | -26.17 |  |
| MSN6 | -25.93 | -21.12 | -27.74 | -28.04 | -29.44 | -20.15 | -10.73 | -22.90 | -26.23 |  |
| MSN7 | -26.00 | -22.79 | -27.73 | -27.94 | -29.77 | -19.89 | -10.29 | -22.41 | -26.17 |  |
|  |  |  |  |  |  |  |  |  |  |  |
| MLN1 | -25.94 | -17.81 | -28.53 | -29.94 | -30.90 | -20.03 | -11.47 | -26.32 | -26.68 |  |
| MLN2 | -23.96 | -17.71 | -29.05 | -29.36 | -29.69 | -19.70 | -10.80 | -21.75 | -26.20 |  |
| MLN3 | -27.36 | -22.36 | -27.51 | -27.77 | -29.75 | -18.97 | -10.46 | -22.46 | -25.71 |  |
| MLN4 | -27.90 | -22.21 | -27.13 | -26.95 | -29.32 | -17.93 | -9.12 | -22.58 | -25.54 |  |
| MLN5 | -27.87 | -21.59 | -27.26 | -26.75 | -28.94 | -17.14 | -8.47 | -21.64 | -25.41 |  |
| MLN6 | -27.60 | -21.18 | -27.27 | -26.63 | -28.99 | -17.74 | -8.87 | -22.68 | -25.39 |  |
| MLN7 | -27.43 | -23.88 | -27.19 | -27.11 | -29.39 | -18.07 | -8.69 | -22.36 | -25.79 |  |
|  |  |  |  |  |  |  |  |  |  |  |
| MAN1 | -23.82 | -18.58 | -27.63 | -25.45 | -27.28 | -17.01 | -8.38 | -20.43 | -23.40 |  |
| MAN2 | -24.31 | -20.68 | -27.36 | -25.64 | -27.71 | -16.83 | -8.43 | -19.88 | -23.64 |  |
| MAN3 | -24.93 | -22.87 | -27.75 | -26.08 | -28.72 | -16.91 | -8.59 | -20.55 | -23.93 |  |
| MAN4 | -24.99 | -19.80 | -27.82 | -26.53 | -26.81 | -16.45 | -8.79 | -20.18 | -23.77 |  |
| MAN5 | -21.81 | -17.76 | -28.22 | -24.69 | -25.56 | -15.87 | -7.89 | -19.38 | -23.00 |  |
| MAN6 | -22.03 | -16.71 | -27.70 | -24.06 | -25.36 | -15.82 | -7.09 | -19.61 | -22.66 |  |
| MAN7 | -22.73 | -17.37 | -28.44 | -24.95 | -26.39 | -16.10 | -8.61 | -20.78 | -23.21 |  |
|  |  |  |  |  |  |  |  |  |  |  |
| MCN1 | -25.47 | -26.83 | -27.80 | -26.12 | -28.91 | -16.05 | -8.86 | -19.95 | -23.79 |  |
| MCN2 | -23.57 | -23.69 | -27.39 | -25.58 | -28.13 | -15.03 | -8.17 | -18.72 | -23.80 |  |
| MCN3 | -24.95 | -24.15 | -27.42 | -24.79 | -27.36 | -15.31 | -7.24 | -19.61 | -23.85 |  |
| MCN4 | -23.86 | -21.73 | -28.21 | -23.85 | -25.31 | -15.68 | -7.71 | -20.28 | -23.43 |  |
| MCN5 | -22.71 | -18.67 | -28.65 | -23.53 | -24.42 | -13.90 | -6.30 | -18.87 | -23.13 |  |
| MCN6 | -25.16 | -21.71 | -27.83 | -23.63 | -25.36 | -14.96 | -6.93 | -20.37 | -23.43 |  |

δ13C (‰ ) (VPDB)

| Time (weeks) | Ala | Gly | Leu | Val | Pro | Thr | Glx | Phe |
| --- | --- | --- | --- | --- | --- | --- | --- | --- |
| -22 | -21.3666 | -15.3633 | -27.0207 | -26.8923 | -16.6698 | -7.58885 | -19.4537 | -24.6599 |
| -22 | -21.4175 | -15.628 | -27.2146 | -27.3783 | -16.8752 | -7.36052 | -18.9024 | -24.4604 |
| -20 | -23.256 | -15.7672 | -27.2142 | -27.5294 | -17.2873 | -7.61166 | -20.2076 | -24.4663 |
| -19 | -21.7484 | -13.9744 | -27.6934 | -27.3084 | -17.5637 | -8.19651 | -19.1058 | -24.5556 |
| -17 | -21.8763 | -15.0407 | -26.8832 | -27.2255 | -17.4269 | -8.35891 | -19.7032 | -24.4807 |
| -3 | -21.3961 | -17.0584 | -25.2396 | -27.0311 | -16.5364 | -7.673 | -22.8451 | -23.4058 |
| 0 | -20.9792 | -17.7858 | -24.7591 | -26.171 | -16.5826 | -7.77238 | -19.5138 | -23.1483 |
| 4 | -22.9703 | -20.1234 | -25.2802 | -27.203 | -16.8105 | -7.64843 | -20.9029 | -23.4859 |
| 19 | -22.1451 | -17.2967 | -25.5106 | -26.9372 | -16.8563 | -8.20256 | -19.8226 | -23.1584 |

Fig. S2 Amino acid *δ*^13^C for MOM 1

δ13C (‰ ) (VPDB)

| Time (weeks) | Ala | Gly | Leu | Val | Pro | Thr | Glx | Phe |
| --- | --- | --- | --- | --- | --- | --- | --- | --- |
| -4 | -22.2858 | -19.1544 | -27.1496 | -28.1429 | -17.3422 | -8.98815 | -20.3715 | -25.0595 |
| 5 | -24.4967 | -19.3922 | -27.9606 | -28.0898 | -17.0515 | -8.72921 | -23.2343 | -25.4903 |
| 32 | -20.6114 | -17.6547 | -23.8795 | -24.4997 | -15.2819 | -8.52522 | -19.6713 | -22.0585 |
| 68 | -21.3836 | -16.7387 | -24.0538 | -24.6641 | -15.589 | -8.43761 | -18.7301 | -22.1459 |
| 70 | -21.6243 | -16.9552 | -24.5548 | -25.8714 | -15.887 | -9.08467 | -18.9239 | -22.694 |
| 73 | -24.34 | -20.6 | -25.2 | -27.18 | -16.44 | -9.5 | -20.88 | -23.13 |
| 75 | -24.0636 | -20.14 | -24.971 | -26.8448 | -16.789 | -9.41304 | -20.4513 | -22.7348 |

Fig. S3 Amino acid *δ*^13^C for CHIL 1

δ13C (‰ ) (VPDB)

| Time (weeks) | Ala | Gly | Leu | Val | Pro | Thr | Glx | Phe |
| --- | --- | --- | --- | --- | --- | --- | --- | --- |
| 74 | -24.291 | -17.093 | -29.1562 | -29.4053 | -19.0791 | -9.07117 | -20.9944 | -26.6227 |
| 68 | -26.0055 | -17.6518 | -30.3752 | -30.5014 | -20.0175 | -9.98397 | -22.0125 | -27.2213 |
| 61 | -26.7806 | -21.4307 | -27.9481 | -29.6423 | -19.7231 | -10.903 | -22.727 | -25.0948 |
| 44 | -26.18 | -21.1 | -27.17 | -28.9 | -18.59 | -9.61 | -21.89 | -25.01 |
| 27 | -26.3529 | -21.499 | -28.3972 | -29.8771 | -20.153 | -11.0782 | -23.8318 | -26.1747 |
| 9 | -25.9332 | -21.1154 | -28.0378 | -29.4444 | -20.1499 | -10.7265 | -22.8974 | -26.2278 |
| -12 | -26.0017 | -22.7897 | -27.9419 | -29.7723 | -19.8873 | -10.2869 | -22.4111 | -26.1739 |

Fig. S4 Amino acid *δ*^13^C for MOM 2

δ13C (‰ ) (VPDB)

| Time (weeks) | Ala | Gly | Leu | Val | Pro | Thr | Glx | Phe |
| --- | --- | --- | --- | --- | --- | --- | --- | --- |
| 71 | -25.9425 | -17.8069 | -29.9379 | -30.8964 | -20.032 | -11.4685 | -26.3222 | -26.6829 |
| 57 | -23.9615 | -17.7124 | -29.3602 | -29.6877 | -19.7004 | -10.7976 | -21.7546 | -26.2048 |
| 41 | -27.3579 | -22.3581 | -27.7745 | -29.7496 | -18.9652 | -10.4645 | -22.4593 | -25.7085 |
| 30 | -27.9033 | -22.2114 | -26.9478 | -29.3237 | -17.9255 | -9.11694 | -22.5767 | -25.5438 |
| 21 | -27.8691 | -21.5928 | -26.7479 | -28.9376 | -17.1389 | -8.47062 | -21.642 | -25.4074 |
| 10 | -27.5976 | -21.177 | -26.6286 | -28.9858 | -17.7429 | -8.87439 | -22.6778 | -25.3851 |
| 2 | -27.4256 | -23.8756 | -27.1056 | -29.3928 | -18.0671 | -8.6882 | -22.3634 | -25.7927 |

Fig. S5 Amino acid *δ*^13^C for CHIL 2

δ13C (‰ ) (VPDB)

| Time (weeks) | Ala | Gly | Leu | Val | Pro | Thr | Glx | Phe |
| --- | --- | --- | --- | --- | --- | --- | --- | --- |
| -25 | -23.8183 | -18.5763 | -25.4461 | -27.2838 | -17.009 | -8.38273 | -20.4297 | -23.3996 |
| -20 | -24.3076 | -20.681 | -25.6432 | -27.7141 | -16.8305 | -8.43156 | -19.8766 | -23.6406 |
| -15 | -24.9311 | -22.8675 | -26.0769 | -28.7175 | -16.9107 | -8.58865 | -20.5507 | -23.9264 |
| -8 | -24.9934 | -19.8038 | -26.5294 | -26.8074 | -16.4512 | -8.79313 | -20.1839 | -23.7743 |
| -3 | -21.8134 | -17.7606 | -24.6919 | -25.5632 | -15.8689 | -7.89466 | -19.385 | -23.0045 |
| 1 | -22.0261 | -16.7073 | -24.0561 | -25.3586 | -15.8236 | -7.09458 | -19.614 | -22.6572 |
| 6 | -22.732 | -17.3691 | -24.9502 | -26.3859 | -16.1006 | -8.60535 | -20.7825 | -23.2105 |

Fig. S6 Amino acid *δ*^13^C for MOM 3

δ13C (‰ ) (VPDB)

| Time (weeks) | Ala | Gly | Leu | Val | Pro | Thr | Glx | Phe |
| --- | --- | --- | --- | --- | --- | --- | --- | --- |
| -8 | -25.4721 | -26.8322 | -26.1224 | -28.9103 | -16.0548 | -8.85683 | -19.9465 | -23.7916 |
| 0 | -23.5668 | -23.6924 | -25.5795 | -28.1346 | -15.0261 | -8.16832 | -18.7185 | -23.8013 |
| 4 | -24.9513 | -24.1473 | -24.7929 | -27.3641 | -15.3134 | -7.24064 | -19.6074 | -23.8525 |
| 9 | -23.8583 | -21.7278 | -23.8459 | -25.312 | -15.6791 | -7.70923 | -20.282 | -23.4317 |
| 13 | -22.7117 | -18.6725 | -23.5255 | -24.4224 | -13.8986 | -6.29745 | -18.8669 | -23.1272 |
| 19 | -25.1568 | -21.7067 | -23.6312 | -25.3646 | -14.9564 | -6.92614 | -20.3698 | -23.4294 |

Fig. S7 Amino acid *δ*^13^C for CHIL 3

Table S3. Average *δ*^13^C of bulk keratin samples and EAA, phenylalanine.

| **Participant (n = 6)** | **Avg. *δ*^13^C_bulk_ (‰) (VPDB)** | **Avg. *δ*^13^C_Phe_ (‰) (VPDB)** | **Range *δ*^13^C_Phe_ (‰) (VPDB)** |
| --- | --- | --- | --- |
| MOM 1 (*n* = 9) | –19.1 ± 0.7 | –24.0 ± 0.7 | –24.7 to –23.2 |
| MOM 2 (*n* = 7) | –21.4 ± 0.1 | –26.1 ± 0.8 | –27.2 to –25.0 |
| MOM 3 (*n* = 7) | –18.7 ± 1.0 | –23.4 ± 0.5 | –23.9 to –22.7 |
| CHIL 1 (*n* = 7) | –18.1 ± 0.2 | –23.3 ± 1.4 | –25.5 to –22.1 |
| CHIL 2 (*n* = 7) | ^a^ | –25.8 ± 0.5 | –26.7 to –25.4 |
| CHIL 3 (*n* = 6) | –17.7 ± 0.3 | –23.6 ± 0.3 | –23.9 to –23.1 |
| *Average* (*n = 43*) | –19.0 ± 1.4 | –24.4 ± 1.3 |  |

^a^CHIL 2 did not have enough keratin for both bulk tissue SIA and CSIA

Δ13CGly-Phe (‰) (VPDB)

| δ13CPhe (‰) (VPDB) | MOM 1 |
| --- | --- |
| -24.6599 | 9.29664 |
| -24.4604 | 8.83247 |
| -24.4663 | 8.69907 |
| -24.5556 | 10.5812 |
| -24.4807 | 9.44001 |
| -23.4058 | 6.34742 |
| -23.1483 | 5.36244 |
| -23.4859 | 3.36251 |
| -23.1584 | 5.86178 |

| δ13CPhe (‰) (VPDB) | CHIL 1 |
| --- | --- |
| -25.0595 | 5.90507 |
| -25.4903 | 6.09807 |
| -22.0585 | 4.40377 |
| -22.1459 | 5.40719 |
| -22.694 | 5.73881 |
| -23.13 | 2.53 |
| -22.7348 | 2.59484 |

| δ13CPhe (‰) (VPDB) | MOM 2 |
| --- | --- |
| -26.6227 | 9.52971 |
| -27.2213 | 9.56946 |
| -25.0948 | 3.66414 |
| -25.01 | 3.91 |
| -26.1747 | 4.67568 |
| -26.2278 | 5.11241 |
| -26.1739 | 3.38418 |

| δ13CPhe (‰) (VPDB) | CHIL 2 |
| --- | --- |
| -26.6829 | 8.87597 |
| -26.2048 | 8.4924 |
| -25.7085 | 3.35035 |
| -25.5438 | 3.33242 |
| -25.4074 | 3.8146 |
| -25.3851 | 4.20812 |
| -25.7927 | 1.91713 |

| δ13CPhe (‰) (VPDB) | MOM 3 |
| --- | --- |
| -23.3996 | 4.8233 |
| -23.6406 | 2.95965 |
| -23.9264 | 1.05887 |
| -23.7743 | 3.97053 |
| -23.0045 | 5.24386 |
| -22.6572 | 5.94984 |
| -23.2105 | 5.84141 |

| δ13CPhe (‰) (VPDB) | CHIL 3 |
| --- | --- |
| -23.7916 | -3.04056 |
| -23.8013 | 0.108918 |
| -23.8525 | -0.294758 |
| -23.4317 | 1.70392 |
| -23.1272 | 4.45472 |
| -23.4294 | 1.72278 |

Fig. S8 Keratin Δ^13^C_Gly-Phe_ versus *δ*^13^C_Phe_ for fingernail samples of mother-infant dyads. Aquatic diets typically show higher *δ*^13^C_Gly_, reflecting higher trophic levels due to greater isotopic fractionation in marine environments (Larsen et al. 2013), while *δ*^13^C_Phe_ remain relatively constant in all consumers. This allows Δ^13^C_Gly-Phe_ to effectively differentiate between aquatic and terrestrial protein sources. Individuals consuming marine protein exhibit a higher Δ^13^C_Gly-Phe_ (12.0 ± 1.9‰) compared to those consuming C_3_ (5.1 ± 1.8‰) and C_4_ terrestrial diets (4.0 ± 1.6‰) (Corr et al. 2005). This figure presents wide intra-individual variability of –3.0 to 10.6‰

**References**

Bean, W. B. (1974). Nail growth: 30 years of observation. *Archives of Internal Medicine, 134*(3), 497-502.

Corr, L. T., Sealy, J. C., Horton, M. C., & Evershed, R. P. (2005). A novel marine dietary indicator utilizing compound-specific bone collagen amino acid *δ*^13^C values of ancient humans. *Journal of Archaeological Science, 32*(3)*,* 321-330.

Fogel, M. L., Tuross, N., & Oswley, D. W. (1989). Nitrogen isotope tracers of human lactation in modern and archaeological populations. *Carnegie Year Books*, *88*, 111-117.

Fuller, B. T., Fuller, J. L., Harris, D. A., & Hedges, R. E. (2006). Detection of breastfeeding and weaning in modern human infants with carbon and nitrogen stable isotope ratios. *American Journal of Physical Anthropology*, *129*(2), 279-293.

Goldsmith, L. A. (1991). *Physiology, Biochemistry and Molecular Biology of the Skin*. Oxford University Press: Oxford.

Hillman, R. W. (1955). Fingernail growth in the human subject: Rates and variations in 300 individuals. *Human Biology*, *27*(4), 274.

Kandil, E. (1972). Accurate measurement of nail growth. *International Journal of Dermatology, 11*(1), 54-56.

Kootker, L. M., Ammer, S. T., Davies, G. R., & Lehn, C. (2024). Isotopic analysis of formula milk reveals potential challenges in geolocating bottle-fed babies. *Scientific Reports*, *14*(1), 1-9.

Larsen, T., Ventura, M., Andersen, N., O’Brien, D. M., Piatkowski, U., & McCarthy, M. D. (2013). Tracing carbon sources through aquatic and terrestrial food webs using amino acid stable isotope fingerprinting. *PloS ONE*, *8*(9), e73441.

Mora, A., Pacheco, A., Roberts, C., & Smith, C. (2018). Pica 8: Refining dietary reconstruction through amino acid *δ*^13^C analysis of tendon collagen and hair keratin. *Journal of Archaeological Science*, *93*(1), 94-109.

Mora, A., Smith, C., Standen, V. G., & Arriaza, B. T. (2022). Bulk and amino acid isotope analyses of hair detail adult diets and infant feeding practices among pre-and post-maize populations of the northern Chilean coast of the Atacama Desert. *Journal of Anthropological Archaeology, 67*(1), 101435.

O'Connell, T. C., Hedges, R. E., Healey, M. A., & Simpson, A. H. R. (2001). Isotopic comparison of hair, nail and bone: Modern analyses. *Journal of Archaeological Science, 28*(11), 1247-1255.

Silverman, S. N., Phillips, A. A., Weiss, G. M., Wilkes, E. B., Eiler, J. M., & Sessions, A. L. (2022). Practical considerations for amino acid isotope analysis. *Organic Geochemistry*, *164*, 104345.
