# Supplementary material for: Amino acid stable carbon isotopes in nail keratin illuminate breastfeeding and weaning practices of mother – infant dyads
Source: Amino Acids. 2025 Jan 30;57(1):13. doi: 10.1007/s00726-024-03425-2 (PMC11782432; doi:10.1007/s00726-024-03425-2)
Supplement: Supplementary file 1 — Supplementary Material 1 [file 726_2024_3425_MOESM1_ESM.pdf]

Title: Amino Acid Stable Carbon Isotopes in Nail Keratin Illuminate Breastfeeding and Weaning Practices of Mother – Infant Dyads

Journal Name: Amino Acids

Authors: *Hana Salahuddin[1], Andrea L. Waters-Rist[1] (0000-0003-1807-3171), Fred J. Longstaffe[2] (0000-0003-4103-4808)

[1]Anthropology, Western University, London, Canada, [2]Earth Sciences, Western University, London, Canada

*Corresponding Author: hsalahu@uwo.ca

Online Resource 2

Dietary and Health Survey

Project Title: Diet, Stress and Everything Else in the Maternal Reproduction and Infant Feeding Ecology. Using Compound Specific Isotope Analysis of Amino Acids for the Reconstruction of Breastfeeding and Weaning Practices.

Principal Investigator: Dr. Andrea Waters-Rist
awaters8@uwo.ca
Department of Anthropology
Western University, London

Student Co-investigator: Hana Salahuddin
hsalahu@uwo.ca
(647)-862-4045
Department of Anthropology
Western University, London

Health and Diet Questionnaire - Mothers

Participant ID: ____________________

Age:
Height:
Weight before pregnancy:
Weight at 1st trimester:
Weight at 2nd trimester:
Weight at 3rd trimester:
Weight after pregnancy:

Did you experience any notable periods of illness or health complication during your pregnancy or after? If so, please briefly describe what you experienced and provide the approximate time (e.g., one month after conception) and duration.

____

____

____

____

____

Do you have any allergies or dietary restrictions?

____

____

Are you currently following a special diet (e.g., low fat, low salt)? If yes, what diet are you on and how long have you been on it?

____

____

How would you describe your diet?

☐ Omnivorous (diverse diet containing both plant and meat)

☐ Vegan (abstaining from the use of animal products)

☐ Lacto-vegetarian (abstaining from meat and eggs, but eating dairy products)

☐ Ovo-lacto-vegetarian (a vegetarian eating eggs and dairy products)

☐ Pescatarian (abstaining from meat, but eating seafood)

☐ Semi-vegetarian (heavy reliance on a vegetarian diet, with occasional consumption of meat)

☐ Other, explain:____

How often do you eat meat (such as poultry, beef, pork, goat, and lamb, and not including fish or seafood) in a week?

☐ Never

☐ Rarely

☐ 1 – 2 times

☐ More than 3 times

☐ Everyday

How often do you eat fish or shellfish in a week? If you consume fish or shellfish, please specify what fish/shellfish you eat regularly:____

- ☐ Never
- ☐ Rarely
- ☐ 1 – 2 times
- ☐ More than 3 times
- ☐ Everyday

Select foods that you eat regularly (at least once a week):

- ☐ Chicken
- ☐ Beef
- ☐ Pork
- ☐ Other meats, if yes please specify:______________________
- ☐ Fish or shellfish, if yes please specify: ___________________________
- ☐ Alternative meat products, if yes please specify:__________________________
- ☐ Beans (e.g. tofu, chickpeas, green, kidney, black, lima, string beans)
- ☐ Peas
- ☐ Lentils
- ☐ Fruits (e.g. apple, banana, orange, grape, berries, melons, lemon/lime, avocado; includes fruit juices, excludes pineapple)
- ☐ Pineapple
- ☐ Root vegetables (e.g. onion, beet, carrot, potato)
- ☐ Leaf and leaf stalk vegetables (e.g. lettuce, cabbage, spinach, brussels sprouts)
- ☐ Other vegetables (e.g., peppers, tomato, celery, broccoli, cauliflower, garlic, cucumber, mushrooms)
- ☐ Olives (including olive oil)
- ☐ Corn and corn-based products
- ☐ Nuts and nut-based products
- ☐ Diary (e.g. milk, yogurt, cheese)
- ☐ Eggs
- ☐ Grains (e.g. rice, bread, couscous, quinoa, pasta)

☐ Other plant-based foods (if yes, please specify): ______

Select foods that you eat occasionally (at least once a month):

☐ Chicken

☐ Beef

☐ Pork

☐ Other meats, if yes please specify:_____________________

☐ Fish or shellfish, if yes please specify: __________________________

☐ Alternative meat products, if yes please specify:_________________________

☐ Beans (e.g. tofu, chickpeas, green, kidney, black, lima, string beans)

☐ Peas

☐ Lentils

☐ Fruits (e.g. apple, banana, orange, grape, berries, melons, lemon/lime, avocado; includes fruit juices, excludes pineapple)

☐ Pineapple

☐ Root vegetables (e.g. onion, beet, carrot, potato)

☐ Leaf and leaf stalk vegetables (e.g. lettuce, cabbage, spinach, brussels sprouts)

☐ Other vegetables (e.g., peppers, tomato, celery, broccoli, cauliflower, garlic, cucumber, mushrooms)

☐ Olives (including olive oil)

☐ Corn and corn-based products

☐ Nuts and nut-based products

☐ Diary (e.g. milk, yogurt, cheese)

☐ Eggs

☐ Grains (e.g. rice, bread, couscous, quinoa, pasta)

☐ Other plant-based foods (if yes, please specify): ______

Did your diet change during or after pregnancy? If yes, please explain (include food aversions and cravings):

____

Did your appetite (more/less hungry) change during or after pregnancy? If yes, please explain:

Are there any other factors that affected your dietary choices during or after pregnancy?

Health and Diet Questionnaire – Children

Child's ID:_______________________

Height at the time of birth:
Height at 6 months:
Height at 12 months:
Height at 18 months:

Weight the time of birth:
Weight at 6 months:
Weight at 12 months:
Weight at 18 months:

Did your child experience any notable period of illness or poor health during early childhood (0 – 5 years)? If so, please explain what they experienced, when it occurred, and how long it lasted?

____

____

Does your child have any allergies or dietary restrictions?

____

____

Did your child at any point follow a special diet (e.g., low fat, low salt)? If yes, what diet were they on and what was their age when it began and ended?

____

____

Did you exclusively breastfeed your child (e.g. only provide breastmilk) for any length of time?
☐ No ☐ Yes.
If yes, how old was your child when you ended exclusive breastfeeding (introduced other liquids or solids)?____
Did you provide your child with breastmilk alternatives such as formula? ☐ No ☐ Yes.
If yes, explain when you began to include these in your child's diet, how often they were fed breastmilk alternatives, and until what age the breastmilk alternatives were consumed.

____

____

____

When did you begin weaning your child off breastmilk? How often would you breastfeed during this period (noting that the frequency may have changed over time)? When did you completely stop breastfeeding?

____

____

____

Does your child drink animal milk (e.g., cow, goat milk) or consume dairy products? If yes, at what age did they begin consuming these foods and how often per day would they consume them?

____

____

____

Does your child drink animal milk alternatives such as soy, almond or oat milk or consume vegan imitation dairy products? If yes, at what age did they begin consuming these foods and how often per day would they consume them?

____

____

____

What foods did you incorporate into your child's diet during this weaning period on a regular basis (i.e. once a week)?

☐ Chicken

☐ Beef

☐ Pork

☐ Other meats, if yes please specify:______________________

☐ Fish or shellfish, if yes please specify: ____________________________

☐ Alternative meat products, if yes please specify:___________________________

☐ Beans (e.g. tofu, chickpeas, green, kidney, black, lima, string beans)

☐ Peas

☐ Lentils

☐ Fruits (e.g. apple, banana, orange, grape, berries, melons, lemon/lime, avocado; includes fruit juices, excludes pineapple)

☐ Pineapple

☐ Root vegetables (e.g. onion, beet, carrot, potato)

☐ Leaf and leaf stalk vegetables (e.g. lettuce, cabbage, spinach, brussels sprouts)

☐ Other vegetables (e.g., peppers, tomato, celery, broccoli, cauliflower, garlic, cucumber, mushrooms)

☐ Olives (including olive oil)

- ☐ Corn and corn-based products
- ☐ Nuts and nut-based products
- ☐ Diary (e.g. milk, yogurt, cheese)
- ☐ Eggs
- ☐ Grains (e.g. rice, bread, couscous, quinoa, pasta)
- ☐ Other plant-based foods (if yes, please specify): ______

What does your child's post-weaning diet include on a regular basis?

- ☐ Chicken
- ☐ Beef
- ☐ Pork
- ☐ Other meats, if yes please specify:_____________________
- ☐ Fish or shellfish, if yes please specify: __________________________
- ☐ Alternative meat products, if yes please specify:_________________________
- ☐ Beans (e.g. tofu, chickpeas, green, kidney, black, lima, string beans)
- ☐ Peas
- ☐ Lentils
- ☐ Fruits (e.g. apple, banana, orange, grape, berries, melons, lemon/lime, avocado; includes fruit juices, excludes pineapple)
- ☐ Pineapple
- ☐ Root vegetables (e.g. onion, beet, carrot, potato)
- ☐ Leaf and leaf stalk vegetables (e.g. lettuce, cabbage, spinach, brussels sprouts)
- ☐ Other vegetables (e.g., peppers, tomato, celery, broccoli, cauliflower, garlic, cucumber, mushrooms)
- ☐ Olives (including olive oil)
- ☐ Corn and corn-based products
- ☐ Nuts and nut-based products
- ☐ Diary (e.g. milk, yogurt, cheese)
- ☐ Eggs

- ☐ Grains (e.g. rice, bread, couscous, quinoa, pasta)
- ☐ Other plant-based foods (if yes, please specify): ______

How often does your child eat foods sweetened with natural sugars (i.e., sugarcane) in a week?

- ☐ Never
- ☐ Rarely
- ☐ 1 – 2 times
- ☐ More than 3 times
- ☐ Everyday

How often does your child eat meat (such as poultry, beef, pork, goat, and lamb, and not including fish or seafood) in a week?

- ☐ Never
- ☐ Rarely
- ☐ 1 – 2 times
- ☐ More than 3 times
- ☐ Everyday

How often does your child eat fish or shellfish in a week? If he/she consumes fish or shellfish, please specify what fish/shellfish:____

- ☐ Never
- ☐ Rarely
- ☐ 1 – 2 times
- ☐ More than 3 times
- ☐ Everyday

Thank you for your participation! ☺
